# Molecular epidemiology and strain diversity of circulating feline Calicivirus in Thai cats

**DOI:** 10.3389/fvets.2024.1377327

**Published:** 2024-06-03

**Authors:** Kannika Phongroop, Jatuporn Rattanasrisomporn, Chutchai Piewbang, Sahatchai Tangtrongsup, Anudep Rungsipipat, Somporn Techangamsuwan

**Affiliations:** ^1^Department of Pathology, Faculty of Veterinary Science, Chulalongkorn University, Bangkok, Thailand; ^2^Animal Virome and Diagnostic Development Research Unit, Faculty of Veterinary Science, Chulalongkorn University, Bangkok, Thailand; ^3^Faculty of Veterinary Medicine, Chiang Mai University, Chiang Mai, Thailand; ^4^Companion Animal Clinical Sciences, Faculty of Veterinary Medicine, Kasetsart University, Bangkok, Thailand

**Keywords:** cats, feline calicivirus, phylogenetic analysis, physicochemical property, strain diversity, Thailand

## Abstract

Feline calicivirus (FCV) is a significant viral pathogen causing upper respiratory tract and oral diseases in cats. The emergence of the virulent systemic FCV variant (VS-FCV) has raised global concern in the past decade. This study aims to explore the epidemiology, genetic characterization, and diversity of FCV strains circulating among Thai cats. Various sample types, including nasal, oral, and oropharyngeal swabs and fresh tissues, were collected from 184 cats across different regions of Thailand from 2016 to 2021. Using reverse transcription real-time polymerase chain reaction (RT-qPCR), FCV infection was investigated, with additional screening for feline herpesvirus-1 (FHV-1) by qPCR. The detection rates for FCV, FHV-1, and co-infection were 46.7, 65.8, and 31.5%, respectively. Significantly, the odds ratio (OR) revealed a strong association between the detection of a single FCV and the presence of gingivostomatitis lesions (OR: 7.15, 95% CI: 1.89–26.99, *p* = 0.004). In addition, FCV detection is notably less likely in vaccinated cats (OR: 0.22, 95% CI: 0.07–0.75, *p* = 0.015). Amino acid sequence analysis based on the VP1 major capsid protein gene of the 14 FCV-Thai (FCV-TH) strains revealed genetic diversity compared to the other 43 global strains (0 to 86.6%). Intriguingly, a vaccine-like FCV variant was detected in one cat. In summary, this study provides insights into the epidemiology and molecular characteristics of FCV diversity within the Thai cat population for the first time. The identification of unique physicochemical characteristics in the capsid hypervariable region of some FCV-TH strains challenges previous hypotheses. Therefore, further exploration of vaccine-like FCV variants is crucial for a comprehensive understanding and to improve viral prevention and control strategies.

## 1 Introduction

Feline upper respiratory tract disease (FURTD) remains a prevalent clinical condition affecting both kittens and adult cats. The primary viral agents causing FURTD are feline calicivirus (FCV) and feline herpesvirus type 1 (FHV-1) (1). FCV, in particular, is a significant viral pathogen associated with FURTD and oral diseases in cats. Clinical manifestations of FCV infection include sneezing, nasal and ocular discharge, conjunctivitis, as well as oral issues like glossitis, gingivostomatitis, and faucitis. While FCV infections in cats typically present mild-to-moderate symptoms that are self-limiting, instances of severe pneumonia, abortion, and acute febrile lameness syndrome linked to FCV have been increasingly reported (1, 2). Additionally, there are reports of a lethal form of severe systemic FCV infection caused by a virulent systemic FCV (VS-FCV) being documented worldwide (3–8).

FCV, belonging to the family *Caliciviridae*, genus *Vesivirus*, is a positive-sense, single-stranded, non-enveloped RNA virus with a size of approximately 7.6–7.7 kb (9–11). The FCV genome contains three major open reading frames (ORFs): ORF1 – ORF3. ORF1 encodes non-structural proteins [p5.6, p32, 2C-like helicase (NTPase), p30, 3C-like protease (Pro), and RNA-dependent RNA polymerase (RdRp)], and a viral protein (VPg) (12). ORF2 encodes the leader capsid (LC) and VP1 major capsid proteins, while ORF3 encodes the VP2 minor capsid protein. The regions between the RdRp and ORF2 are crucial genetic hotspots, particularly in terms of recombinant events, contributing significantly to the genomic evolution within the *Caliciviridae* family (13).

Based on amino acid sequence alignment and antigenic analysis, ORF2 can be divided into six distinct regions (A–F) (14). Region A is associated with the LC proteins, while regions B, D, and F are relatively conserved. Regions C and E are considered highly divergent among FCV isolates (10). Region E is particularly immunodominant and comprises three parts: 5′ hypervariable region (5’HVR-E), conserve central region (Cons-E), and 3′ hypervariable regions (3’HVR-E) (14, 15).

The VP1 major capsid protein monomer consists of three structural domains: the N-terminal arm (NTA), shell (S) domains, and protrusion (P) domains. The P domain, a flexible protruding structure, plays a crucial role in virus-host receptor interaction and antigenic diversity (10, 15). Within the P domain, there are the P1 and P2 subdomains, with P2 containing the 5’HVR-E antigenic region. This region, located on the outermost surface of the viral capsid, contains two distinct linear epitopes recognized by both neutralizing and non-neutralizing monoclonal antibodies (10). Additionally, specific physicochemical properties of amino acid residues at positions 438, 440, 448, 452, 455, 465, and 492 in the HVR-E region have been suggested to be associated with VS-FCV pathotype variance (16).

While FCV is acknowledged as a highly variable RNA virus with considerable genetic diversity (17, 18), it is commonly referred to as a single serotype (19, 20). Previous studies have emphasized the potential cross-protective effects of FCV vaccines (21–24). However, various FCV strains, such as F9, FCV255, G1, and 431, are frequently employed in commercial vaccine products (25), raising concerns about the emergence of vaccine-resistant strains (20) and potential failures in vaccination (19, 26). In Thailand, feline vaccination protocols align with the guidelines of the American Animal Hospital Association (AAHA) and the American Association of Feline Practitioners (AAFP) (27). Despite the widespread use of commercial vaccines, both killed and live-attenuated, the FCV detection rate in Thai cats remains above 50% (28), and the genetic characteristics of circulating FCV strains in Thailand are not well understood. Therefore, this study aimed to elucidate the molecular epidemiology, genetic and amino acid profiles, and diversities of circulating FCV strains in Thai cats from 2016 to 2021. Additionally, we assessed genetic variability, phylogenetic relationships, and the physicochemical properties of amino acid residues in the HVR-E regions among FCV Thai strains (FCV-TH), other wild-type strains, and vaccine strains obtained from the GenBank database.

## 2 Materials and methods

### 2.1 Sample collection and ethics

The study utilized a cross-sectional design, employing a convenient sampling of 184 cats from diverse regions in Thailand, spanning from January 2016 to July 2021. Data collection encompassed general signalments such as age, vaccination history, and presenting clinical signs. The inclusion criteria involved symptoms related to FURTD, including ocular discharge, sneezing, coughing, and oral diseases, with a specific focus on gingivostomatitis and oral ulceration. Cats exhibiting respiratory issues associated with nasal deformity, asthma, and neoplasia were excluded from the study.

Out of the 184 sampled cats, swab samples were obtained from 163 live cats while fresh tissue (FT) samples were collected from 21 cats that had succumbed to respiratory illnesses. For the living cats, nasal swabs (NS), oropharyngeal swabs (OS), and rectal swabs (RS) were collected from each cat and individually immersed in 1% sterile phosphate buffer saline (PBS). Additionally, FT samples, including the tongue, buccal mucosal, trachea, lung, gastrointestinal tract, brain, and all lymph nodes, were obtained from deceased cats submitted to the Department of Pathology, Faculty of Veterinary Science, Chulalongkorn University, as part of routine necropsy procedures. Swab and FT samples were stored at −80°C until the extraction of viral nucleic acid. All procedures were conducted under the approval of the Chulalongkorn University Animal Care and Use Committee (No. 1631002).

### 2.2 Viral nucleic acid extraction and FCV and FHV-1 detection using real-time polymerase chain reaction (qPCR)

Supernatants from swab and homogenized FT samples using 1% PBS and Tissue Rupture (Qiagen GmbH, Hilden, Germany) underwent viral nucleic extraction through the QIAamp^®^*cador* Pathogen Mini Kit (Qiagen GmbH, Hilden, Germany), following the manufacturer’s recommendation. The concentration and quality of the extracted nucleic acid were determined using a NanoDrop Lite Spectrophotometer (Thermo Fisher Scientific Inc., Waltham, MA, United States). The extracted samples were then kept at −80°C until further use. Complimentary DNA (cDNA) was constructed using an Omniscript^®^ RT Kit (*Qiagen* GmbH, Hilden, *Germany*) in accordance with the manufacturer’s guidelines and previously described protocol (29). The constructed cDNA samples were stored at −20°C until assayed.

The presence of FCV was determined using qPCR with newly designed specific primers targeting a 122-bp region in the non-structural (NS) gene within ORF1 (FCV_NS F2/R2; Supplementary Table S1). The qPCR reaction was carried out using KAPA SYBR^®^ FAST qPCR Master Mix (2X) KIT (KAPA BIOSYSTEM, Sigma-Aldrich^®^, Modderfontein, South Africa) following the manufacturer’s instruction, with the reactions conducted on a Rotor-Gene^®^ Q instrument (Qiagen GmbH, Manheim, Germany). The cycling conditions comprised an initial denaturation at 95°C for 3 min, followed by 40 cycles at 95°C for 3 s, 60°C for 20 s, and 64°C for 20 s. The melting analysis was subsequently performed using Rotor-Gene Q software version 2.3.1 (Qiagen^®^ GmbH, Manheim, Germany). All samples were processed in duplicate.

For FHV-1 screening, we utilized self-designed specific primers (FHV-gB F/R) targeting a 176-bp region in the glycoprotein B (gB) genome of FHV-1 (Supplementary Table S1). The qPCR reaction was conducted using the same reagent and condition as described above. Commercially available vaccines (Nobivac^®^ 1-HCPCh, Intervet Inc., Ohama, NE, U.S.A), which contain FCV and FHV-1, were included as positive controls. The negative control of the reactions was employed using distilled water. Positive qPCR assays for both FCV and FHV-1 were defined by a Ct value less than 35.

After qPCR analysis, the selected positive amplicons of FCV (122 bp) and FHV-1 (176 bp) were resolved in 2% (w/v) gel electrophoresis, purified using the NucleoSpin^®^ Extract II kit (Macherey Nagel, Düren, Germany), and then submitted for bi-directional Sanger’s sequencing (Macrogen Inc., Incheon, South Korea) to confirm the presence of FCV and FHV-1 nucleic acids.

### 2.3 FCV genome sequencing, genetic characterization, and phylogenetic analysis of FCV

FCV-positive samples with a Ct value less than 25 underwent multiple conventional RT-PCR amplifications to achieve complete genome sequencing. Degenerated primer sets were designed based on multiple alignments of various FCV strains available in the GenBank database (Supplementary Table S1). Briefly, the RT-PCR reactions were performed using a Qiagen^®^ OneStep RT-PCR kit (Qiagen^®^, Hilden, Germany) in a 50 μL reaction volume, consisting of, a mixture of QIAGEN^®^ OneStep RT-PCR Enzyme Mix, 10 mM of dNTP in 5x QIAGEN^®^ OneStep RT-PCR Buffer, 10 μM final concentration of each primer, and 10 μL of the template. Thermocycler conditions included 50°C for 30 min for the RT step, followed by an initial denaturation at 95°C for 15 min, 40 cycles of 94°C for 10 s, 58°C for 1 min, and 72°C for 2 min, and then a final extension at 72°C for 10 min. The PCR products were visualized using 1% (w/v) gel electrophoresis, purified, and submitted for Sanger sequencing as per the aforementioned protocols.

The obtained nucleotide sequences of the FCV-TH strain were assembled and compared with previously published FCV strains available in the GenBank database using BioEdit Sequencing Alignment Editor Version 7.2.5. The FCV Urbana strain (Accession no. L40021) served as a reference for determining amino acid positions. Phylogenetic analysis and pairwise genetic distance were conducted using MEGA X (30). The phylogeny was established following the Bayesian information criterion and constructed using the maximum likelihood (ML) method. Statistical support was assessed through 1,000 bootstrapped replicates, considering values greater than 70 as significant. Substitution models for the phylogenetic tree were selected using the best-fit model algorithm embedded in the Bioedit software. The resulting phylogenetic analysis was visualized using FigTree v.1.4.4. A 20% genetic distance threshold between VP1 capsid sequences was applied to define distinct strains (11, 17, 31).

### 2.4 Genetic recombination and selective pressure analyses of FCV

The Recombination Detection Program (RDP) version 4.101 was utilized to identify potential recombination events within both the whole genome and the VP1 region of FCV-TH strains (32). Analytical methods, including RDP, GENECONV, BootScan, MaxChi, Chimera, SiScan, and 3Seq, were employed with default settings. Potential recombination events were indicated when the program illustrated positive results in more than four analytical methods (33).

To assess nucleotide substitutions in the FCV genome indicative of rapid adaptation, selective pressure analysis was authenticated using the codon-based approach through the Datamonkey Adaptive Evolution Server (34, 35). This involved analyzing the ratio of nonsynonymous (dN) to synonymous (dS) substitutions per site, utilizing maximum likelihood phylogenetic reconstruction and the general reversible nucleotide substitution model. Various models, including Single-likelihood ancestor counting (SLAC), Fixed-effects likelihood (FEL), Mixed Effect Model Evolution (MEME), and Fast Unconstrained Bayesian AppRoximation (FUBAR) algorithms, were employed for non-neutral selection analysis. The significance threshold for SLAC, FEL, and MEME was set at a *p*-value ≤0.1, while FUBAR used a posterior probability of 0.9. The rate of dN and dS within an individual codon was estimated with a Bayes factor of 50, categorizing codons based on posterior probabilities: dN/dS > 1 for positive diversifying selection, dN/dS = 1 for neutral mutation, and dN/dS < 1 for negative selection.

### 2.5 Statistical analysis

The overall prevalence of FCV detection in Thailand was determined, and associations between FCV and FHV-1 detection, clinical presentations, and vaccination history were assessed using Fisher’s exact or Pearson chi-square tests, as applicable. Odds ratios (OR) and 95% confidence intervals (95% CI) for associated variables were estimated through multinomial logistic regression. A significance level of less than 0.05 (*p* < 0.05) was considered statistically significant. Statistical analyses were conducted using STATA software release 16.1 (Stata Corp., College Station, Texas, United States).

## 3 Results

### 3.1 Occurrence of FCV and FHV-1 circulating in Thai cats

Among the 184 cats studied, 53 were clinically healthy (28.8%), 111 had signs related to FURTD (60.3%), and 42 exhibited oral diseases (22.8%). Notably, 22 cats (12%) suffered from both respiratory and oral diseases (Supplementary Table S2).

The prevalence rates were 46.7% (86/184) for FCV, 65.8% (121/184) for FHV-1, and 31.5% (58/184) for co-detection. Detection rates varied across regions and among the sampled cats in this study (Table 1). In non-respiratory cats, FCV, FHV-1, and co-detection rates were 17.8% (13/73), 41.1% (30/73), and 20.6% (15/73), respectively. Moreover, FCV positive detection in non-gingivostomatitis cats was 42.3% (60/142). Based on qPCR-positive results for both viruses, FCV was predominantly in OS at 46.5% (74/159), followed by NS at 35.5% (49/138), FT at 23.8% (5/21), and RS at 5.6% (2/36). FHV-1 detection was highest in OS at 57.2% (91/159), NS at 42% (58/138), FT at 66.7% (14/21), and RS at 33.3% (12/36). Details on vaccination status are provided in Supplementary Table S2.

**Table 1:** Prevalence of feline calicivirus (FCV), feline herpesvirus type-1 (FHV-1), and co-detection from 184 cats from different regions in Thailand between 2016 and 2021.

Region / Province (n)	FCV positivity (n)	FHV-1 positivity (n)	Co-detected positivity (n)
North (33) Chiang Mai Tak Phayao Nakhon Sawan Uttaradit	24.2% (8)	69.7% (23)	21.2% (7)
Northeast (17) Khon Kaen Sakon Nakhon	35.3% (6)	94.1% (16)	29.4% (5)
East (4) Chonburi	25% (1)	25% (1)	0 (0)
South (11) Phuket	100% (11)	81.8% (9)	81.8% (9)
Central (119) Bangkok and vicinity Saraburi	50.4% (60)	60.5% (72)	31.1% (37)
Overall prevalence (184)	46.7% (86)	65.8% (121)	31.5% (58)

Remarkably, one FCV qPCR-positive cat (no 165) exhibited clinical signs resembling VS-FCV infection, including generalized edema, ulcerative dermatitis at the upper lip, and severe acute respiratory illness (Supplementary Table S2), while others displayed classical symptoms or remained asymptomatic.

### 3.2 Associations between FCV, FHV-1, and co-detection with clinical variables

The data included general signalments such as age (ranging from 1 to 154 months), vaccination history (within 1 year prior to sampling), and presenting clinical signs. Associations between viral detection and clinical variables, including signs of oral and respiratory diseases and vaccination history against FCV and FHV-1 within a year before sampling, were analyzed. Single FCV detection was statistically significant with gingivostomatitis (*p* = 0.004), while single FHV-1 detection was significant in cats that showed respiratory illness within the last 3 months before sample collection (*p* = 0.009; Table 2). Moreover, the multivariable multinomial logistic regression result indicated that single FCV-detected cats had a higher risk of developing gingivostomatitis lesions (OR = 7.15, 95% CI: 1.89–26.99) than other infections. Meanwhile, the presented respiratory sign was not associated with either single or double viral detection (Table 2).

**Table 2:** The multivariable-adjusted multinomial logistic regression analysis of variables associated with feline calicivirus (FCV) and feline herpesvirus-1 (FHV-1) detection from different regions in Thailand between 2016 and 2021 (*n* = 162).

Variables^a^	FCV			FHV-1			FCV and FHV-1 co-detection		
	Odds ratios	95% CI^*^	*p* value^#^	Odds ratios	95% CI^*^	*p* value^#^	Odds ratios	95% CI^*^	*p* value^#^
Gingivostomatitis	**7.15**	**1.89–26.99**	**0.004**	1.30	0.39–4.32	0.664	1.11	0.31–3.99	0.829
Recent respiratory illness	1	0.37–2.73	~ 1.00	0.88	0.39–2.00	0.761	2.29	0.95–5.54	0.065
Presenting respiratory illness within the last 3 months	0.61	0.18–2.05	0.421	**0.25**	**0.09–0.70**	**0.009**	1.03	0.40–2.66	0.065
Vaccination within one year	**0.22**	**0.07–0.75**	**0.015**	1.28	0.43–3.86	0.655	**0.21**	**0.08–0.55**	**0.002**

a Variables of clinical presentations: oral sign showed gingivostomatitis; respiratory sign displayed chronic upper respiratory tract problems such as nasal discharge and sneezing.^*^95% confidence interval.^#^Accepting significant *p*-value as < 0.05.

A noteworthy association was also evident between previous vaccination status and the positive detection of FCV and FCV/FHV-1 (*p* < 0.05). Vaccinated cats exhibited a significantly lower likelihood of viral detection, approximately 4.5 times lesser than unvaccinated cats, for both single FCV detection (OR = 0.22, 95% CI: 0.07–0.75) and both FCV/FHV-1 detection (OR = 0.21, 95% CI: 0.08–0.55). Interestingly, this correlation was not observed in cats that tested positive solely for FHV-1 (Table 2).

### 3.3 Sequencing and phylogenetic analysis

This study successfully obtained one full-length complete genome sequence, 13 partial RdRp (aa 354–502), 15 full-length VP1, and 13 VP2 coding sequences (Supplementary Table S2). The 7,671 nucleotides (nt) complete coding sequence of one FCV-TH strain (KP361/THA/2021: cat no.165; Accession no. MZ542330) was achieved.

**Figure 1:**
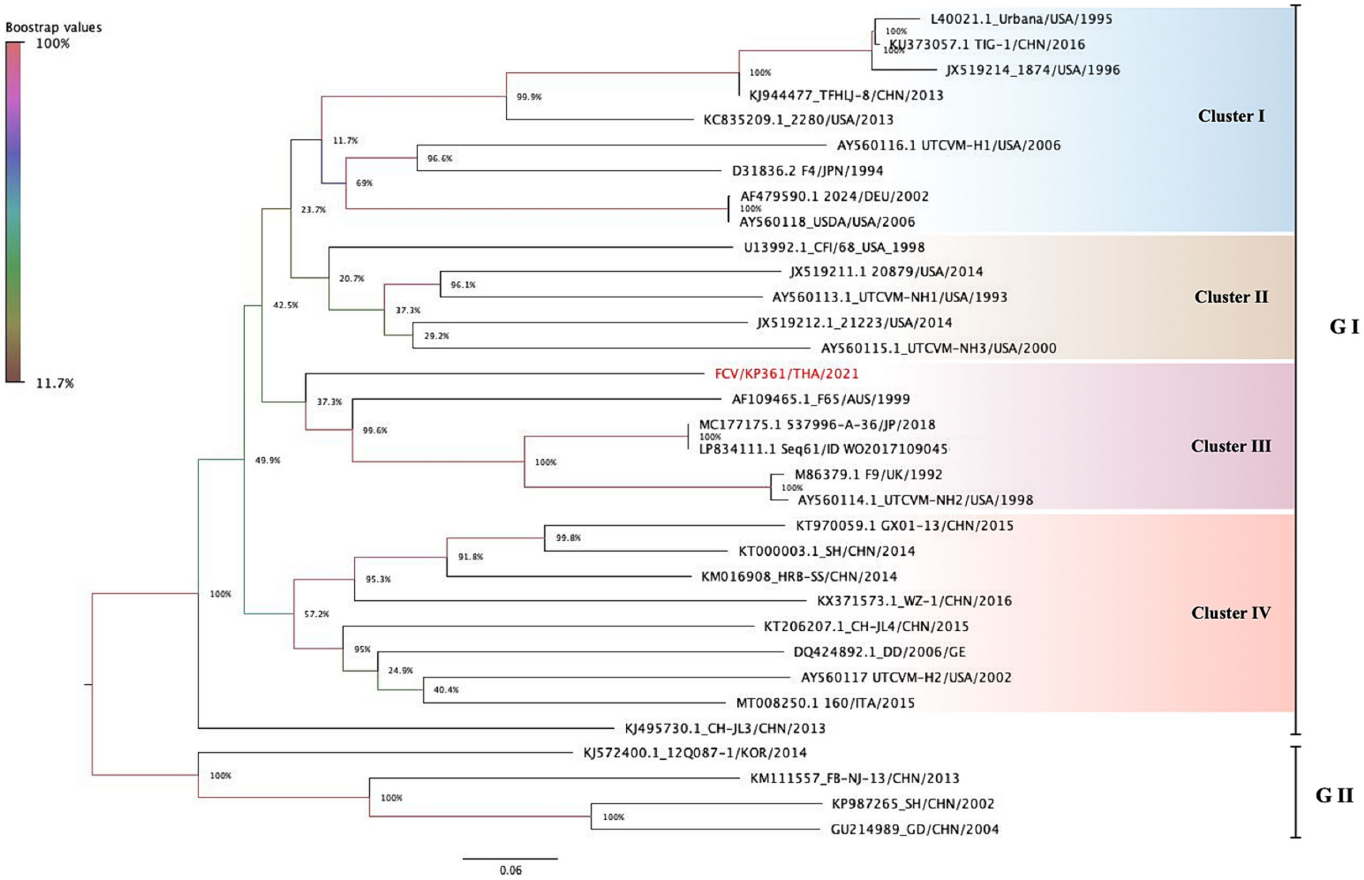
The phylogenetic tree is constructed based on 33 complete nucleotide sequences of feline calicivirus (FCV). The tree is created using the maximum likelihood method with General Time Reversible (GTR) model. The phylogeny test is encouraged by 1,000 bootstrapped replicates, and the bootstra*p* value is significantly considered when greater than 70. FCV-TH strain is labeled with a red circle. Interclade divergence of FCV-TH to others is represented by a p-distance threshold limited as <20 percent divergence for a single strain.

Phylogenetic analysis based on the complete coding genome sequence of FCV revealed that the KP361/THA/2021 strain clustered within cluster III of genotypes I (GI), grouped with five other strains (strain F65; Accession no. AF109465, strain JP 2018537996-A/36; Accession no. MC177175, strain Sequence 61 from Patent WO2017109045; Accession no. LP834111, strain F9; Accession no. M86379, and strain UTCVM-NH2; Accession no. AY560114). The nucleotide distance among them ranged from 49.3 to 55.6% (Figure 1).

Overall, the nucleotide identity of the KP361/THA/2021 strain ranged from 39.1–79.3% compared to the other 32 complete genome sequences available in the GenBank database (Table 3). The highest identity, 79.3%, closely matched two FCV strains documented as vaccine strains in the GenBank database (strains JP 2018537996-A/36; Accession no. MC177175 and strain Sequence 61 from Patent WO2017109045; Accession no. LP834111).

**Table 3:** Nucleotide (nt) and deduced amino acid (aa) identity between 14 feline calicivirus Thai (FCV-TH) strains and other 43 FCV strains deposited in the GenBank database.

	Whole genome		Full-length VP1		P2 subdomain		HVR-E	
	nt (%)	aa (%)	nt (%)	aa (%)	nt (%)	aa (382–550)	nt (%)	aa (426–520)
Between FCV-TH strain and other strains in GenBank	39.1–79.3	N/A	34.6–94.9	79.0–96.4	44.7–92.7	66.6–90.5	32.2–90.1	60.0–84.3%
Within group (FCV-TH strain)	N/A	N/A	61.4–99.7	84.0–99.8	42.4–99.6	70.7–99.4	36.1–100	63.5–100%

N/A, Not applicable.

**Figure 2:**
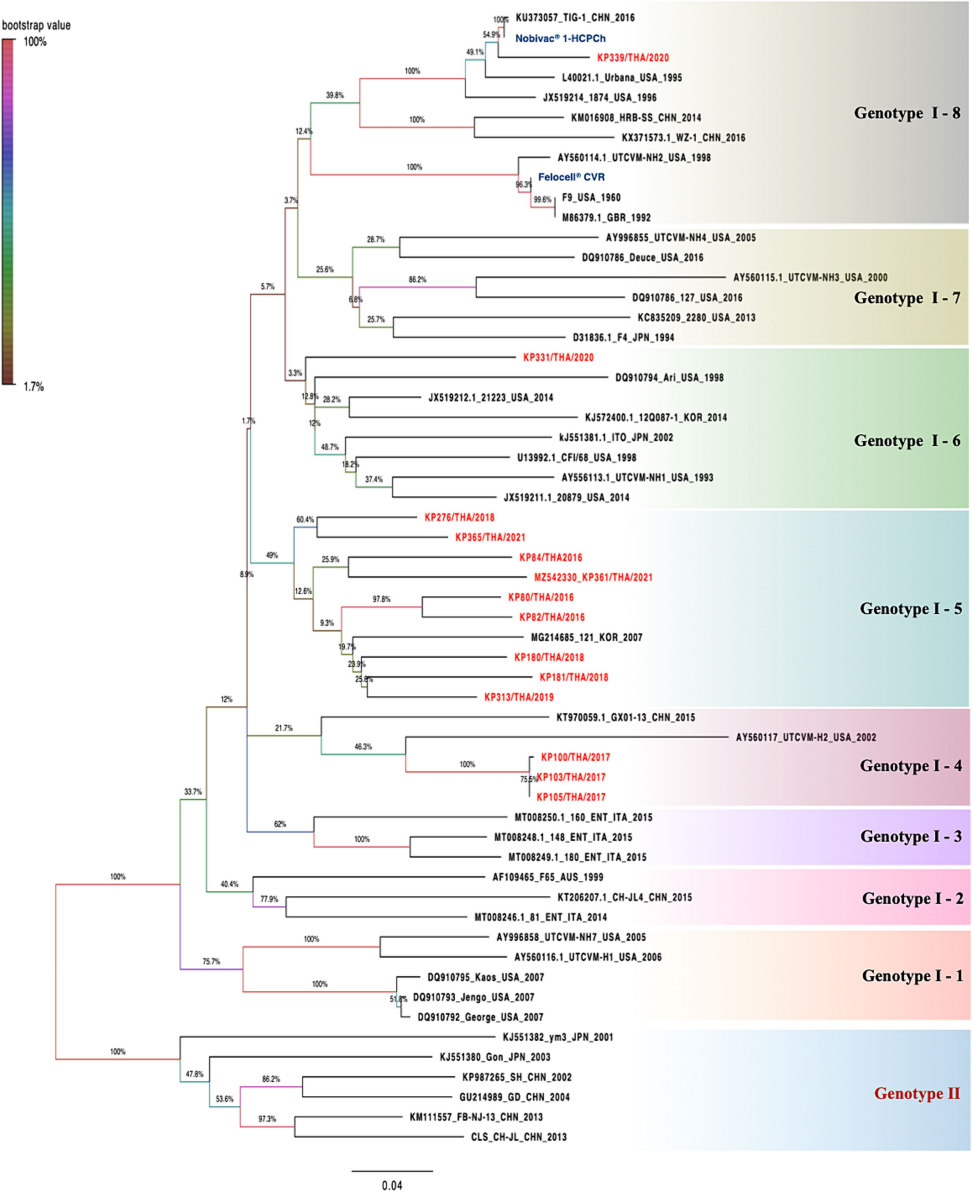
The phylogenetic is constructed based on 57 deduced amino sequences of full-length VP1 of feline calicivirus (FCV). The trees were constructed based on deduced amino acids using the maximum likelihood method with Le Gascuel 2008 model. The statistical support supplied with bootstrapping of 1,000 replicates and significantly considered when a bootstrap value is greater than 70. FCV-TH strains were presented in red letters. The pairwise p-distance of each group was shown in each box by a limited threshold as <20 percent divergence for a single strain.

The deduced amino acid identity and constructed phylogenetic tree based on the amino acid sequences of the obtained 14 full-length VP1 sequences were compared with 43 other FCV-VP1 sequences available in the GenBank database (Figure 2; Supplementary Figures S1, S2). The homology between the FCV-TH strains and the global strains ranged from 79.0 to 96.4%. Additionally, the VP1 sequence among FCV-TH strains derived from this study shared amino acid identity within the range of 84 to 99.8% (Table 3).

Within the phylogenetic relationship based on the VP1 domain of FCV, which was categorized into two main genotypes (GI and GII), all FCV-TH strain sequences were grouped within the GI genotype. Upon analyzing the genetic distance between groups of amino acids, we observed that the genetic distance between GI and GII was approximately 17.6–20.3%. The GI genotype could be further divided into eight genetic subtypes based on the branch length of the phylogenetic tree. Most FCV-TH strains (9/14) formed their subtype (GI-5), accompanied by one FCV strain from Korea (strain 121; Accession no. MG214685). The genetic distance within these subtypes ranged from 8.3 to 21.3% (Figure 2).

Interestingly, three additional FCV-TH strains (KP100/THA/2017; cat no.59, KP103/THA/2017; cat no.62, and KP105/THA/2017, cat no.55; Accession no. OM982625-OM982627, respectively) were grouped within the same genetic subtype (GI-4) together with VS-FCV strains from China and the United States (strain GX01-13; Accession no. KT970059 and strain UTCVM-H2; Accession no. AY560117). They were genetically shared with VS-FCV strain UTCVM-H2 as their common ancestor. Furthermore, one FCV-TH strain (KP339/THA/2020; cat no.151; Accession no. OM982632) revealed the genetic topology related to two commercially available vaccine sequences (Nobivac^®^ 1-HCPCh; Intervet Inc., Ohama, NE, U.S.A, and Felocell^®^ CVR; Ser No. 464968B, Zoetis^©^, Lincoin, NE, United States), both clustered into the same subtype (GI-8; Figure 2). The amino acid divergence between the VP1 capsid protein sequence of KP339/THA/2020 and the vaccine strains resulted in 4.8 and 24.5% divergence with the Nobivac^®^ and Felocell^®^ CVR, respectively.

Within the VP1 amino acid properties, the association between amino acid residues at positions 438, 440, 448, 452, 455, 465, and 492, and the pathotype identification [VS-FCV pathotype or classical (CLD) pathotype (16)] was assessed. Three out of 14 FCV-TH strains (KP100/THA/2017, KP103/THA/2017, and KP105/THA/2017) displayed physicochemical properties associated with the VS-FCV pathotype (Table 4), even though those cats did not present any severe respiratory symptom. However, it was noteworthy that one FCV-TH strain (KP361/THA/2021), which exhibited typical clinical signs of the VS-FCV pathotype, did not show any significant residue positions that aligned with the pathotype differentiation criteria outlined in previous publications (16) (Table 4).

**Table 4:** Physicochemical property of remarkable amino acid residue position on hypervariable region E (HVR-E) of VP1 major capsid protein gene hypothesized to differentiate between classical FCV and VS-FCV pathotype.

	aa 438	aa 440	aa 448	aa 452	aa 455	aa 465	aa 492
Strains	Hydrophilic aliphatic	Not small	Polar positive charge	Not small	Not negative charge	Polar	Small
VS-FCV^*^ (21 sequences)	V_10_, T_11_	S_3_, K,**E**_**4**_, Q_6_, G_7_	A_3_, P_2_, G_2_,**R**, K_11_, E, G	D_7_,**E**_**14**_	E, D_6_, N,**T**_**7**_, I_2_, M_3_, S	**S**_**16**_, G_5_	**V**_**19**_, R, I
CLD-FCV^#^ (26 sequences)	T_21_, V_3_, A_2_	S_8_, Q_3_, D, K, G_13_	A_19_, R_3_, P_2_, G, K	D_24_, E_2_	D_13_, S_2_, T_5_, A_2_, G_2_, V, I	S_8_, G_18_	V_13_, K_3_, R_6_, L_2_, I_2_
KP80/THA/2016	T	G	A	D	D	G	**V**
KP82/THA/2016	T	G	A	D	D	G	I
KP84/THA/2016	T	**E**	A	D	D	G	V
**KP100/THA/2017**	T	**E**	**R**	**E**	**T**	**S**	**V**
**KP103/THA/2017**	T	**E**	**R**	**E**	**T**	**S**	**V**
**KP105/THA/2017**	T	**E**	**R**	**E**	**T**	**S**	**V**
KP180/THA/2018	T	G	A	D	E	G	**V**
KP181/THA/2018	T	G	P	D	D	G	**V**
KP276/THA/2018	T	G	A	D	D	G	**V**
KP313/THA/2019	T	D	A	D	D	G	**V**
KP331/THA/2020	T	G	A	D	**T**	**S**	**V**
KP339/THA/2020	T	R	A	D	H	G	**V**
KP361/THA/2021	T	G	A	D	D	G	I
KP365/THA/2021	T	S	S	N	D	G	**V**

Residues in bold signify amino acids that match the configuration of the VS-FCV.^*^VS-FCV refers to the virulent systemic feline calicivirus.^#^CLD-FCV refers to the classical feline calicivirus.

### 3.4 Recombinant and selective pressure analyses

**Figure 3:**
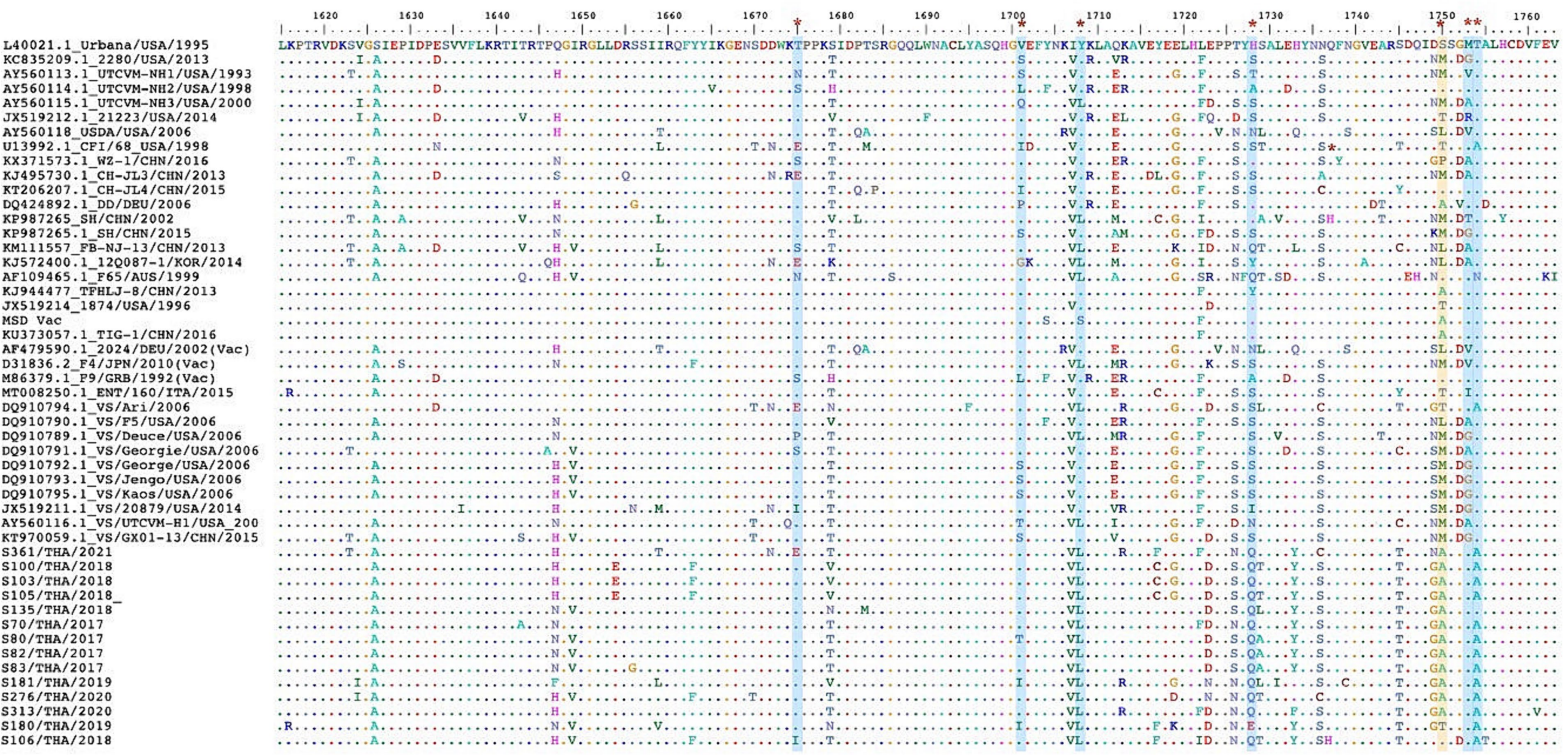
The multiple deduced amino acid sequence alignment of partial RNA dependent RNA polymerase (RdRp) protein (aa1615-1763). The sequence comprises 49 sequences, including 14 FCV-TH strains and 35 sequences from the GenBank database. The seven positions (red asterisk) of evidentially positive episodic selection by MEME (blue strip) were shown. One deduced amino acid positive had demonstrated both evidentially of positive episodic selection by MEME and positive pervasive/purifying selection by FEL (yellow strip).

The *in-silico* analysis conducted to detect evidence of genetic recombination did not reveal any recombinant events within the FCV-TH variants. The dN/dS ratio was obtained from the whole genome, the full-length VP1 gene, and the partial RdRp gene of various available FCV strains using a codon-based analysis. Overall, the FCV sequences had undergone negative selection, as obtained results presented in SLAC, FEL, and MEME with a dN/dS ratio < 1. However, the MEME test revealed potential evidence of 24 amino acid sites of VP1 that had undergone episodic positive selection. Three locations of amino acid residues that demonstrated the positive selection evidence by MEME stood on 3 of 7 remarkable amino acid residues (448, 455, and 492) with a *p-value* threshold of 0.1 (Table 5). Likewise, the partial RdRp gene sequence (aa 1,615–1737) exhibited positive episodic selection by MEME on four deduced amino acid sites (1,675, 1701, 1708, and 1728). Additionally, FCV-TH strains displayed distinct deduced amino acid characteristics in residues at the 3′ end of ORF1, including Q1728, Y1733, T1745, G1749, A1750, and A1754 (Figure 3).

**Table 5:** Evidence of positive and negative selection using various detection methods.

FCV genome	Selection pressure analysis	SLAC	FEL	MEME
VP 1 gene	Positive selection	0	0	24^a^
	Negative selection	593	616	-
	Overall dN/dS	0.058	0.058	0.058
Partial RdRp (aa 1,615–1737)	Positive selection	0	1^b^	7^c^
	Negative selection	110	113	-
	Overall dN/dS	0.065	0.054	0.054

Amino acid diversifying selection site.^a^MEME: 7, 29, 31, 82, 91, 120, 366, 396, 400, 408, 411, 441, 448, 455, 483, 492, 497, 500, 502, 506, 507, 542, 625, 637.^b^FEL: 1750.^c^MEME: 1675, 1701, 1708, 1728, 1750, 1753, 1754.

## 4 Discussion

Feline viral upper respiratory tract infection, primarily caused by FCV and FHV-1, is commonly diagnosed in Thai cats in clinical settings. Commercially available vaccines derived from various FCV strains, such as the F9 strain (22, 36), FCV-255 strain (37), FCV strain 21 (38), and FCV-G1, are widely used in Thailand. Notably, some of these vaccines are formulated as bivalent, providing protection against multiple FCV strains, thereby enhancing the breadth of immune defense. However, this study reveals a relatively high prevalence of FCV (46.7%) circulating in Thai cats, indicating a higher FCV detection rate compared to other countries where prevalence ranges from 9.2 to 36%^1^ (17, 20, 39, 40). Previous studies have noted that the prevalence of FCV usually alters according to the number of cats in a household, showing 10 to 40% with low density in large populations and with high density in the house, respectively (40–43). Similar to this study, a majority of the samples were obtained from cats residing in multi-cat households, with over half of the population being free-living cats (data not shown).

This study reveals not only a high prevalence of FCV detection but also a significantly higher likelihood of viral detection, approximately 4.5 times, in unvaccinated cats compared to their vaccinated counterparts in both contexts of single FCV and dual FCV/FHV-1 detections. These results suggest a considerable level of efficacy in the vaccines, indicating robust protectivity within the Thai cat population. However, it is crucial to acknowledge the ongoing controversy surrounding the efficacy of these available vaccines. Numerous publications have raised questions and concerns regarding their effectiveness, especially the prevention of highly virulent FCV strains (4, 7, 41). This issue underscores the need for continual research and investigations to assess the performance of FCV vaccines, ensuring the most robust and reliable protection for feline populations. However, this study did not investigate the other potential causative agents of feline respiratory tract infections, including Feline Infectious Peritonitis virus (FIP), *Mycoplasma felis*, *Chlamydophila felis*, and *Bordetella bronchiseptica.* Co-infections with these agents can exacerbate symptoms and pose challenges for treatment.

This study has also disclosed a significant correlation between the detection of single FCV infection and the presence of gingivostomatitis lesions. However, only 22.8% of the samples were derived from cats exhibiting gingivostomatitis, aligning with findings in previous studies (44–47). Despite these connections, the link between the FCV RNA load and the severity of the disease in FCV-positive cats with feline chronic gingivostomatitis remains unestablished (48). It is noteworthy that, in contrast to a multitude of publications, the FCV antigen has not been identified within the chronic gingivostomatitis lesions of cats through immunohistochemistry (49). Despite this, certain hypotheses have surfaced, suggesting that feline chronic gingivostomatitis might stem from an immune-mediated reaction to FCV (50). This hypothesis remains intriguing and warrants further in-depth investigation.

Our study has contributed to the ongoing discussion about the classification of FCV genotypes. Previous publications have presented conflicting perspectives on whether FCV can be classified into one or two genotypes (51–55). In this study, phylogenetic trees based on the nucleotide and amino acid sequences of the VP1 gene illustrated two distinct genotypes of FCV. The FCV-TH strain was placed in the GI genotype in both topologies, and most of them formed their genetic subtype when the phylogenetic tree was constructed using the amino acid sequence of the VP1 gene. This finding suggests that the circulation of FCV in Thai cats is currently identifiable only within the GI genotype.

The identification of an FCV-TH strain (KP339/THA/2020) showing phylogenetic relatedness to a commercial vaccine strain (Nobivac^®^ 1-HCPCh, Intervet Inc., Ohama, NE, United States) is noteworthy. The cat associated with this strain had been vaccinated within the past year but not within the 3 months preceding the sample collection. Despite this recent vaccination, the cat consistently experienced chronic respiratory tract issues. A previous study has reported the successful detection of FCV vaccine strain (e.g., F9) with genetic divergence in the HVR-E region of less than 3% in cats up to 70 days after vaccination (56). The hypothesis is that cats might accidentally ingest spilled vaccine material from their fur, leading to inadvertent infection with the FCV vaccine strain (56). There have also been reports of vaccine strain (F9)-like viruses in general cat populations (18, 57, 58). In this study, the genetic divergence of the detected FCV-TH strain (KP339/THA/2020) from the associated commercial vaccine strain, calculated based on the HVR-E region, was greater than 20%. This divergence suggests that it may not be a direct FCV vaccine variant, despite their close phylogenetic relationship. The hypothesis is that the cat might have been exposed to an FCV vaccine-like field strain, causing chronic respiratory tract infection. This situation emphasizes the importance of thoroughly investigating the evolution of vaccine-derived FCV and its potential role in causing vaccine-related respiratory tract disease in the future.

The identification of three samples (KP100/THA/2017, KP103/THA/2017, KP105/THA/2017) from unvaccinated kittens with mild clinical respiratory symptoms, showing homology in their VP1 capsid protein amino acid sequence and a relationship to a VS-FCV strain (strain UTCVM-H2; Accession No. AY560117) in the phylogenetic tree, is intriguing. The analysis of amino acid physicochemical properties of the HVR-E region in their VP1 gene revealed results inconsistent with a previous report highlighting seven notable residues potentially segregating between classical and VS-FCV strains (16). Contrary to the expected results, the sequences of these three samples matched the seven key amino acid residues represented by the VS-FCV strain. In contrast, the sequence of one FCV-TH strain (KP361/THA/2021), derived from a clinically associated VS-FCV cat, demonstrated different characteristics. It clustered with other circulating FCV-TH strains in subtype GI-5 when the phylogenetic tree was constructed based on the VP1 gene sequence. Moreover, it grouped within the same cluster as five other global strains in the phylogenetic tree based on the whole genome nucleotide sequence, three of which were documented in the GenBank database as vaccine strains. Additionally, none of the physicochemical criteria used for pathotype differentiation of KP361/THA/2021 matched the seven noteworthy amino acid residues associated with the VS-FCV pathotype. Following the observation of this study, three out of seven remarkable amino acid residues (448, 455, and 492), significantly associated with pathotype differentiation (16), have undergone episodic positive selection is intriguing. This suggests that these specific amino acid positions might be under selective pressure, potentially contributing to the evolution and adaptation of FCV strains. The interest in verifying whether geographic variations and vaccination background contribute to distinct characteristics of VS-FCV or classical strains in different regions is valid. This could shed light on how FCV evolves and adapts in response to various environmental and immunological pressures. Further investigation into the genetic and phenotypic variations of FCV strains, considering factors such as geographic location, vaccination practices, and host populations, is warranted. This could provide valuable insights into the factors influencing the evolution and pathogenicity of FCV, which, in turn, can inform better strategies for vaccine development and control measures.

Previous study has indicated regions that were frequently identified as antibody evasion point mutations, located at amino acid positions 441, 448, 449, and 455 of the VP1 gene (59). Moreover, the mutation-prone region spanning amino acids position 445–451, recognized as the neighborhood of the neutralizing epitope (10), further emphasizes the significance of this region in the virus’s interaction with the host immune system.

While no recombination evidence was found in the Thai variance from this study, the unique divergence observed in some amino acid residues in the RdRp gene (Q1728, T1745, and A1754) among FCV-TH strains is noteworthy. Moreover, evidence of episodic positive/diversifying selection on amino acid position S1750A, predominantly in FCV-TH strain, further emphasized the potential influence of selective pressure on the genetic diversity of FCV. The higher mutation rate of RdRp, as indicated by the positive selection on amino acid position S1750A, aligns with previous studies highlighting the role of RdRp in increasing genetic diversity and influencing viral population fitness under selective pressures (60).

Our study has some limitations that should be acknowledged. Firstly, the sample size was relatively small, particularly from certain regions or provinces, which may limit the ability to accurately determine the prevalence and conduct a comprehensive analysis of FCV genotypes in Thai cats. Secondly, the lack of previous sequence information on FCV-TH strains and the inability to culture FCV isolates due to funding constraints have made it challenging to obtain complete genomic coding sequences from the limited amount of specimens available. These limitations may affect the completeness and generalizability of our findings.

## 5 Conclusion

This study shed light on the epidemiological status and molecular characteristics of FCV diversity circulating within the Thai cat population, providing a theoretical basis for further research, and improving prevention and control strategies against FCV in Thailand. Notably, as the prevalence of FCV among Thai cats has not been previously documented, this study serves as the inaugural exploration into the molecular makeup, phylogenetic relationships, and distinct physicochemical properties of FCV strains present in Thai cats. This challenges previous hypotheses and suggests that regions with high FCV prevalence experience rapid genomic adaptations to selective environmental forces. The observed amino acid diversity at various phylogenetic levels underscores FCV’s adaptive capabilities within or among species. Further research in Thailand is crucial for developing effective FCV prevention and control strategies, particularly in understanding vaccine-like variants and their evolution in the context of FURTD.

## Data availability statement

The datasets presented in this study can be found in online repositories. The names of the repository/repositories and accession number(s) can be found in the article/Supplementary material.

## Ethics statement

The animal studies were approved by Chulalongkorn University Animal Care and Use Committee (No. 1631002). The studies were conducted in accordance with the local legislation and institutional requirements. Written informed consent was obtained from the owners for the participation of their animals in this study.

## Author contributions

KP: Conceptualization, Data curation, Formal analysis, Investigation, Methodology, Software, Writing – original draft. JR: Resources, Writing – review & editing. CP: Conceptualization, Data curation, Validation, Writing – review & editing. SaT: Data curation, Writing – review & editing. AR: Visualization, Writing – review & editing. SoT: Conceptualization, Funding acquisition, Project administration, Resources, Supervision, Validation, Visualization, Writing – review & editing.

## Footnotes

^1^Wang J, Qian P, Liu C, Cao Y, Chen Y, Sun C, et al. Molecular Investigation of Feline Calicivirus Infection in China 2019–2020. (2021) (http://doi.org/10.21203/rs.3.rs-869342/v1)
