# Supplementary material for: Molecular epidemiology and strain diversity of circulating feline Calicivirus in Thai cats
Source: Front Vet Sci. 2024 Jun 3;11:1377327. doi: 10.3389/fvets.2024.1377327 (PMC11180889; doi:10.3389/fvets.2024.1377327)
Supplement: Supplementary file 1 [file Table_1.docx]

**Supplementary Table S1** Primer sequences for feline calicivirus (FCV) and feline herpesvirus type-1 (FHV-1) detection and full-length FCV genome amplification

| **Primer name** | **Direction** | **Primer Sequence (5'- 3')** | **Nucleotide position^a^** | **Melting**  **Temp** | **Reference** |
| --- | --- | --- | --- | --- | --- |
| FCV_NS F1 | Forward | GTAAAAGAAATTTGAGACAATGTCT | 1 | 51.6 | This article |
| FCV_NS R1 | Reverse | TGTTGATTGGCGGGTAG | 2439 | 52.4 | This article |
| FCV_NS F2 | Forward | GAACTACCCGCCAATCA | 2429^b^ | 52.4 | This article |
| FCV_NS R2 | Reverse | AGCACRYCATATGCGGC | 2550^b^ | 52.4 | This article |
| FCV_NS F3 | Forward | TGGTG**Y**AAGGAGTATGTC | 1931 | 51.6 | This article |
| FCV_NS R3 | Reverse | TCCACCATGATCTAAATTTTACTGCA | 3098 | 61.6 | This article |
| FCV_NS F4 | Forward | TGCAGTAAAATTTAGATCATGGTGGA | 3073 | 61.6 | This article |
| FCV_RdRp F1 | Forward | TATGGTGATGATGGWGTKTAYATGTT | 4778 | 60 | This article |
| FCV_RdRp F2 | Forward | GGYGTGGAGGCGCGGWC | 5237 | 62 | This article |
| FCV_RdRp R | Reverse | CGTHAGCGCAGGTTGAGCACAT | 5335 | 64 | This article |
| FCV_VP1 F1 | Forward | ATGTGCTCAACCTGCGCTAACG | 5314 | 64 | This article |
| FCV_VP1 F2 | Forward | CCCTCAYGTTCTATTTGATGCT | 6066 | 58.4 | This article |
| FCV_VP2 R | Reverse | TGTRTATGAGTAAGGGTCAACC | 7568 | 58.4 | This article |
| FHV_gB F | Forward | CTCGATGGCCCTAGAACGTC | 60008^b^ | 62.5 | This article |
| FHV_gB R | Reverse | AGTCATTGAGGGCACGGAAG | 60183^b^ | 60.5 | This article |

^a^ Nucleotide position based on Accession no. L40021 (for FCV) and MH070336 (for FHV-1)

^b^ Primers used for FCV and FHV-1 detection
