# Supplementary material for: Molecular epidemiology and strain diversity of circulating feline Calicivirus in Thai cats
Source: Front Vet Sci. 2024 Jun 3;11:1377327. doi: 10.3389/fvets.2024.1377327 (PMC11180889; doi:10.3389/fvets.2024.1377327)
Supplement: Supplementary file 2 [file Table_2.docx]

**Supplementary table S2** Demographic data of age, vaccination history, clinical sign presentation, and molecular detection of feline calicivirus (FCV) and feline herpesvirus-1 (FHV-1) in each sample types of 184 studied cats

| **Cat no.^1^** | **Age (Month)** | **Vaccination history^2^** | **Clinical sign presentations^1^** | | **RT-qPCR result^3^** | **Sample types^4^** | | | | **qPCR result^3^** | **Sample types^4^** | | | | **Sequencing^5^** | | | | |
| --- | --- | --- | --- | --- | --- | --- | --- | --- | --- | --- | --- | --- | --- | --- | --- | --- | --- | --- | --- |
|  |  |  | **Oral** | **Respiratory** | **FCV** | **NS** | **OS** | **RS** | **FT** | **FHV-1** | **NS** | **OS** | **RS** | **FT** | **VP1** | **VP2** | **RdRP** | **WGS** | **Strain’s name** |
| 1 | 8 | No | Yes | No | Pos | Pos | Pos |  |  | Neg | Neg | Neg |  |  |  |  |  |  |  |
| 2 | 104 | Yes | Yes | No | Pos | Pos | Pos |  |  | Neg | Neg | Neg |  |  |  |  |  |  |  |
| 3 | 2 | No | Yes | No | Pos | Pos | Pos |  |  | Neg | Neg | Neg |  |  |  |  |  |  |  |
| 4 | 132 | No | Yes | Yes | Pos | Pos | Pos |  |  | Neg | Neg | Neg |  |  |  |  |  |  |  |
| 5 | 24 | No | No | Yes | Neg | Neg | Neg |  |  | Neg | Neg | Neg |  |  |  |  |  |  |  |
| 6 | 120 | Yes | Yes | Yes | Neg | Neg | Neg |  |  | Neg | Neg | Neg |  |  |  |  |  |  |  |
| 7 | 36 | No | Yes | Yes | Pos | Pos | Neg |  |  | Neg | Neg | Neg |  |  |  |  |  |  |  |
| 8 | 84 | Yes | No | No | Neg | Neg | Neg |  |  | Neg | Neg | Neg |  |  |  |  |  |  |  |
| 9 | 4 | No | No | Yes | Pos | Neg | Pos |  |  | Neg | Neg | Neg |  |  |  |  |  |  |  |
| 10 | 4 | Yes | No | No | Neg | Neg | Neg |  |  | Pos | Neg | Pos |  |  |  |  |  |  |  |
| 11 | 11 | Yes | No | No | Neg | Neg | Neg |  |  | Pos | Neg | Pos |  |  |  |  |  |  |  |
| 12 | N/A | No | No | No | Neg | Neg | Neg |  |  | Pos | Neg | Pos |  |  |  |  |  |  |  |
| 13 | N/A | Yes | No | No | Neg | Neg | Neg |  |  | Pos | Neg | Pos |  |  |  |  |  |  |  |
| 14 | N/A | Yes | No | No | Neg | Neg | Neg |  |  | Neg | Neg | Neg |  |  |  |  |  |  |  |
| 15 | 36 | Yes | No | No | Neg | Neg | Neg |  |  | Pos | Neg | Pos |  |  |  |  |  |  |  |
| 16 | 48 | Yes | No | No | Neg | Neg | Neg |  |  | Pos | Neg | Pos |  |  |  |  |  |  |  |
| 17 | 48 | Yes | No | No | Neg | Neg | Neg |  |  | Pos | Neg | Pos |  |  |  |  |  |  |  |
| 18 | 48 | Yes | No | No | Neg | Neg | Neg |  |  | Pos | Neg | Pos |  |  |  |  |  |  |  |
| 19 | 84 | Yes | No | No | Neg | Neg | Neg |  |  | Pos | Neg | Pos |  |  |  |  |  |  |  |
| 20 | 3 | No | No | Yes | Neg | Neg | Neg |  |  | Pos | Neg | Pos |  |  |  |  |  |  |  |

| **Cat no.^1^** | **Age (Month)** | **Vaccination history^2^** | **Clinical sign presentations^1^** | | **RT-qPCR result^3^** | **Sample types^4^** | | | | **qPCR result^3^** | **Sample types^4^** | | | | **Sequencing^5^** | | | | |
| --- | --- | --- | --- | --- | --- | --- | --- | --- | --- | --- | --- | --- | --- | --- | --- | --- | --- | --- | --- |
|  |  |  | **Oral** | **Respiratory** | **FCV** | **NS** | **OS** | **RS** | **FT** | **FHV-1** | **NS** | **OS** | **RS** | **FT** | **VP1** | **VP2** | **RdRP** | **WGS** | **Strain’s name** |
| 21 | 3 | No | No | Yes | Neg | Neg | Neg |  |  | Pos | Neg | Pos |  |  |  |  |  |  |  |
| 22 | 71 | No | Yes | Yes | Pos | Neg | Pos |  |  | Pos | Neg | Pos |  |  |  |  |  |  |  |
| 23 | 36 | Yes | No | No | Neg | Neg | Neg |  |  | Pos | Neg | Pos |  |  |  |  |  |  |  |
| 24 | N/A | Yes | Yes | Yes | Neg | Neg | Neg |  |  | Pos | Neg | Pos |  |  |  |  |  |  |  |
| 25 | N/A | Yes | Yes | Yes | Neg | Neg | Neg |  |  | Pos | Pos | Neg |  |  |  |  |  |  |  |
| 26 | N/A | No | Yes | Yes | Neg | Neg | Neg |  |  | Pos | Pos | Neg |  |  |  |  |  |  |  |
| 27 | N/A | Yes | Yes | No | Neg | Neg | Neg |  |  | Pos | Pos | Pos |  |  |  |  |  |  |  |
| 28 | N/A | Yes | Yes | Yes | Neg | Neg | Neg |  |  | Pos | Pos | Neg |  |  |  |  |  |  |  |
| 29 | N/A | Yes | Yes | Yes | Pos | Pos | Pos |  |  | Pos | Pos | Pos |  |  |  |  |  |  |  |
| 30 | N/A | No | No | Yes | Pos | Neg | Pos |  |  | Pos | Pos | Pos |  |  |  |  |  |  |  |
| 31 | N/A | Yes | Yes | Yes | Pos | Pos | Neg |  |  | Pos | Pos | Pos |  |  |  |  |  |  |  |
| 32 | N/A | Yes | No | Yes | Neg | Neg | Neg |  |  | Pos | Neg | Pos |  |  |  |  |  |  |  |
| 33 | N/A | Yes | Yes | Yes | Neg | Neg | Neg |  |  | Pos | Pos | Pos |  |  |  |  |  |  |  |
| 34 | N/A | Yes | Yes | No | Neg | Neg | Neg |  |  | Neg | Neg | Neg |  |  |  |  |  |  |  |
| 35 | N/A | Yes | Yes | No | Neg | Neg | Neg |  |  | Pos | Neg | Pos |  |  |  |  |  |  |  |
| 36 | N/A | Yes | Yes | No | Neg | Neg | Neg |  |  | Pos | Neg | Pos |  |  |  |  |  |  |  |
| 37 | N/A | Yes | Yes | No | Pos | Neg | Pos |  |  | Pos | Pos | Pos |  |  |  |  |  |  |  |
| 38 | N/A | Yes | Yes | No | Neg | Neg | Neg |  |  | Pos | Pos | Pos |  |  |  |  |  |  |  |
| 39 | N/A | Yes | No | No | Neg | Neg | Neg |  |  | Pos | Pos | Pos |  |  |  |  |  |  |  |
| 40 | 12 | Yes | No | Yes | Pos | Pos | Neg |  |  | Pos | Pos | Neg |  |  |  |  |  |  |  |
| 41 | 36 | Yes | No | No | Pos | Neg | Pos |  |  | Pos | Neg | Pos |  |  |  |  |  |  |  |
| 42 | 12 | No | No | No | Pos | Neg | Pos |  |  | Pos | Pos | Pos |  |  |  |  |  |  |  |
| 43 | 2 | No | Yes | Yes | Pos | Pos | Pos |  |  | Pos | Pos | Neg |  |  |  | Yes | Yes |  | KP70/THA/  2016 |
| **Cat no.^1^** | **Age (Month)** | **Vaccination history^2^** | **Clinical sign presentations^1^** | | **RT-qPCR result^3^** | **Sample types^4^** | | | | **qPCR result^3^** | **Sample types^4^** | | | | **Sequencing^5^** | | | | |
|  |  |  | **Oral** | **Respiratory** | **FCV** | **NS** | **OS** | **RS** | **FT** | **FHV-1** | **NS** | **OS** | **RS** | **FT** | **VP1** | **VP2** | **RdRP** | **WGS** | **Strain’s name** |
| 44 | N/A | No | No | Yes | Pos | Neg | Pos |  |  | Neg | Neg | Neg |  |  |  |  |  |  |  |
| 45 | N/A | No | No | No | Neg | Neg | Neg |  |  | Neg | Neg | Neg |  |  |  |  |  |  |  |
| 46 | N/A | No | No | Yes | Pos | Pos | Pos |  |  | Pos | Neg | Pos |  |  |  |  |  |  |  |
| 47 | N/A | No | No | Yes | Pos | Pos | Pos |  |  | Pos | Neg | Pos |  |  |  |  |  |  |  |
| 48 | N/A | No | No | Yes | Pos | Neg | Pos |  |  | Pos | Neg | Pos |  |  |  |  |  |  |  |
| 49 | N/A | No | No | Yes | Pos | Pos | Pos |  |  | Pos | Neg | Pos |  |  | Yes | Yes | Yes |  | KP80/THA/  2016 |
| 50 | N/A | No | No | Yes | Pos | Neg | Pos |  |  | Pos | Neg | Pos |  |  |  |  |  |  |  |
| 51 | N/A | No | No | Yes | Pos | Pos | Pos |  |  | Pos | Neg | Pos |  |  | Yes |  | Yes |  | KP82/THA/  2016 |
| 52 | N/A | No | No | Yes | Pos | Pos | Pos |  |  | Neg | Neg | Neg |  |  |  |  | Yes |  | KP83/THA/  2016 |
| 53 | N/A | No | No | Yes | Pos | Pos | Pos |  |  | Pos | Neg | Pos |  |  | Yes | Yes |  |  | KP84/THA/  2016 |
| 54 | N/A | No | No | Yes | Pos | Pos | Pos |  |  | Pos | Pos | Pos |  |  |  |  |  |  |  |
| 55 | 5 | No | No | Yes | Pos | Pos | Pos |  |  | Pos | Pos | Pos |  |  | Yes |  | Yes |  | KP105/THA/  2017 |
| 56 | 5 | No | No | Yes | Pos | Pos | Pos |  |  | Pos | Pos | Pos |  |  | Yes |  | Yes |  | KP106/THA/  2017 |
| 57 | N/A | No | No | Yes | Pos | Pos | Pos |  |  | Pos | Neg | Pos |  |  |  |  |  |  |  |
| 58 | N/A | No | No | Yes | Pos | Pos | Pos |  |  | Pos | Neg | Pos |  |  |  |  |  |  |  |
| 59 | 5 | No | No | Yes | Pos | Pos | Pos |  |  | Pos | Neg | Pos |  |  | Yes | Yes | Yes |  | KP100/THA/  2017 |
| 60 | N/A | No | No | Yes | Pos | Pos | Pos |  |  | Pos | Pos | Pos |  |  |  |  |  |  |  |
| 61 | N/A | No | No | Yes | Pos | Pos | Pos |  |  | Pos | Pos | Pos |  |  |  |  |  |  |  |
| 62 | 5 | No | No | Yes | Pos | Pos | Pos |  |  | Pos | Pos | Pos |  |  | Yes |  | Yes |  | KP103/THA/  2017 |
| 63 | N/A | No | No | Yes | Pos | Neg | Pos |  |  | Pos | Pos | Neg |  |  |  |  |  |  |  |
| 64 | N/A | No | No | No | Neg | Neg | Neg |  |  | Neg | Neg | Neg |  |  |  |  |  |  |  |
| 65 | N/A | No | No | No | Neg | Neg | Neg |  |  | Neg | Neg | Neg |  |  |  |  |  |  |  |
| **Cat no.^1^** | **Age (Month)** | **Vaccination history^2^** | **Clinical sign presentations^1^** | | **RT-qPCR result^3^** | **Sample types^4^** | | | | **qPCR result^3^** | **Sample types^4^** | | | | **Sequencing^5^** | | | | |
|  |  |  | **Oral** | **Respiratory** | **FCV** | **NS** | **OS** | **RS** | **FT** | **FHV-1** | **NS** | **OS** | **RS** | **FT** | **VP1** | **VP2** | **RdRP** | **WGS** | **Strain’s name** |
| 66 | N/A | No | No | No | Neg | Neg | Neg |  |  | Neg | Neg | Neg |  |  |  |  |  |  |  |
| 67 | N/A | No | No | Yes | Pos | Pos | Pos |  |  | Pos | Pos | Pos |  |  |  |  |  |  |  |
| 68 | N/A | No | No | Yes | Pos | Pos | Pos |  |  | Pos | Pos | Pos |  |  |  |  |  |  |  |
| 69 | N/A | No | No | Yes | Pos | Pos | Pos |  |  | Pos | Pos | Pos |  |  |  |  |  |  |  |
| 70 | N/A | No | No | Yes | Pos | Pos | Pos |  |  | Pos | Pos | Pos |  |  |  |  |  |  |  |
| 71 | N/A | No | No | Yes | Pos | Pos | Pos |  |  | Pos | Pos | Pos |  |  |  |  |  |  |  |
| 72 | N/A | No | No | Yes | Pos | Pos | Pos |  |  | Pos | Pos | Pos |  |  |  |  |  |  |  |
| 73 | 24 | Yes | No | No | Pos | Pos | Pos |  |  | Pos | Pos | Pos |  |  |  |  |  |  |  |
| 74 | N/A | No | No | Yes | Pos | Pos | Pos |  |  | Pos | Pos | Pos |  |  |  |  | Yes |  | KP135/THA/  2016 |
| 75 | N/A | No | No | No | Pos | Pos | Pos |  |  | Pos | Pos | Pos |  |  |  |  |  |  |  |
| 76 | 29 | Yes | No | No | Pos | Pos | Pos |  |  | Pos | Pos | Pos |  |  |  |  |  |  |  |
| 77 | 8 | Yes | No | No | Pos | Pos | Pos |  |  | Pos | Pos | Pos |  |  |  |  |  |  |  |
| 78 | 12 | No | No | No | Pos | Pos | Pos |  |  | Pos | Pos | Pos |  |  |  |  |  |  |  |
| 79 | 84 | Yes | No | No | Pos | Pos | Pos |  |  | Pos | Pos | Pos |  |  |  |  |  |  |  |
| 80 | N/A | No | No | No | Pos | Pos | Pos |  |  | Pos | Pos | Pos |  |  |  |  |  |  |  |
| 81 | 5 | No | No | No | Pos | Pos | Pos |  |  | Neg | Neg | Neg |  |  |  |  |  |  |  |
| 82 | 24 | No | No | No | Pos | Pos | Pos |  |  | Pos | Neg | Pos |  |  |  |  |  |  |  |
| 83 | N/A | No | Yes | Yes | Pos | Pos | Pos |  |  | Neg | Neg | Neg |  |  |  |  |  |  |  |
| 84 | 156 | No | No | Yes | Pos | Neg | Pos |  |  | Pos | Pos | Pos |  |  |  |  |  |  |  |
| 85 | N/A | Yes | No | No | Pos | Neg | Pos |  |  | Pos | Pos | Pos |  |  |  |  |  |  |  |
| 86 | 96 | No | No | Yes | Neg | Neg | Neg |  |  | Pos | Neg | Pos |  |  |  |  |  |  |  |
| 87 | 96 | No | No | Yes | Neg | Neg | Neg |  |  | Pos | Neg | Pos |  |  |  |  |  |  |  |
| 88 | N/A | Yes | Yes | No | Pos | Pos | Neg |  |  | Pos | Neg | Pos |  |  |  |  |  |  |  |
| **Cat no.^1^** | **Age (Month)** | **Vaccination history^2^** | **Clinical sign presentations^1^** | | **RT-qPCR result^3^** | **Sample types^4^** | | | | **qPCR result^3^** | **Sample types^4^** | | | | **Sequencing^5^** | | | | |
|  |  |  | **Oral** | **Respiratory** | **FCV** | **NS** | **OS** | **RS** | **FT** | **FHV-1** | **NS** | **OS** | **RS** | **FT** | **VP1** | **VP2** | **RdRP** | **WGS** | **Strain’s name** |
| 89 | N/A | Yes | Yes | No | Neg | Neg | Neg |  |  | Pos | Pos | Pos |  |  |  |  |  |  |  |
| 90 | 12 | Yes | No | Yes | Neg | Neg | Neg |  |  | Pos | Pos | Pos |  |  |  |  |  |  |  |
| 91 | 10 | Yes | No | Yes | Neg | Neg | Neg |  |  | Pos | Pos | Pos |  |  |  |  |  |  |  |
| 92 | 36 | Yes | Yes | No | Pos | Neg | Pos |  |  | Neg | Neg | Neg |  |  | Yes | Yes | Yes |  | KP180/THA/  2018 |
| 93 | 107 | No | Yes | No | Pos | Neg | Pos |  |  | Neg | Neg | Neg |  |  | Yes |  | Yes |  | KP181/THA/  2018 |
| 94 | 136 | Yes | No | Yes | Pos | Pos | Pos |  |  | Pos | Pos | Pos |  |  |  |  |  |  |  |
| 95 | 108 | No | No | Yes | Pos | Neg | Pos |  |  | Pos | Neg | Pos |  |  |  |  |  |  |  |
| 96 | 154 | Yes | No | Yes | Pos | Pos | Pos |  |  | Pos | Pos | Neg |  |  |  |  |  |  |  |
| 97 | 93 | Yes | No | Yes | Pos | Pos | Pos |  |  | Pos | Pos | Pos |  |  |  |  |  |  |  |
| 98 | 36 | No | No | Yes | Pos | Neg | Pos |  |  | Pos | Pos | Pos |  |  |  |  |  |  |  |
| 99 | N/A | Yes | No | Yes | Neg | Neg | Neg |  |  | Pos | Pos | Pos |  |  |  |  |  |  |  |
| 100 | 94 | N/A | No | Yes | Neg | Neg | Neg |  |  | Pos | Pos | Neg |  |  |  |  |  |  |  |
| 101 | 12 | N/A | No | Yes | Neg | Neg | Neg |  |  | Pos | Pos | Pos |  |  |  |  |  |  |  |
| 102 | 11 | N/A | No | Yes | Neg | Neg | Neg |  |  | Pos | Pos | Pos |  |  |  |  |  |  |  |
| 103 | N/A | Yes | No | Yes | Neg | Neg | Neg |  |  | Neg | Neg | Neg |  |  |  |  |  |  |  |
| 104 | 120 | Yes | No | Yes | Neg | Neg | Neg |  |  | Pos | Pos | Pos |  |  |  |  |  |  |  |
| 105 | N/A | Yes | No | Yes | Pos | Pos | Pos |  |  | Neg | Neg | Neg |  |  |  |  |  |  |  |
| 106 | 6 | No | Yes | No | Neg | Neg | Neg |  |  | Neg | Neg | Neg |  |  |  |  |  |  |  |
| 107 | N/A | No | No | No | Pos |  | Pos | Neg |  | Pos |  | Neg | Pos |  |  |  |  |  |  |
| 108 | N/A | Yes | No | Yes | Neg |  | Neg | Neg |  | Pos |  | Neg | Pos |  |  |  |  |  |  |
| 109 | N/A | Yes | No | Yes | Neg |  | Neg | Neg |  | Pos |  | Neg | Pos |  |  |  |  |  |  |
| 110 | N/A | Yes | No | Yes | Neg |  | Neg | Neg |  | Pos |  | Neg | Pos |  |  |  |  |  |  |
| 111 | N/A | Yes | No | Yes | Pos |  | Pos | Neg |  | Neg |  | Neg | Neg |  |  |  |  |  |  |
| **Cat no.^1^** | **Age (Month)** | **Vaccination history^2^** | **Clinical sign presentations^1^** | | **RT-qPCR result^3^** | **Sample types^4^** | | | | **qPCR result^3^** | **Sample types^4^** | | | | **Sequencing^5^** | | | | |
|  |  |  | **Oral** | **Respiratory** | **FCV** | **NS** | **OS** | **RS** | **FT** | **FHV-1** | **NS** | **OS** | **RS** | **FT** | **VP1** | **VP2** | **RdRP** | **WGS** | **Strain’s name** |
| 112 | N/A | Yes | No | Yes | Neg |  | Neg | Neg |  | Pos |  | Neg | Pos |  |  |  |  |  |  |
| 113 | N/A | Yes | No | Yes | Neg |  | Neg | Neg |  | Pos |  | Neg | Pos |  |  |  |  |  |  |
| 114 | N/A | Yes | No | Yes | Neg |  | Neg | Neg |  | Neg |  | Neg | Neg |  |  |  |  |  |  |
| 115 | N/A | Yes | No | Yes | Neg | Neg | Neg | Neg |  | Pos | Pos | Pos | Pos |  |  |  |  |  |  |
| 116 | 1 | Yes | No | No | Neg |  | Neg | Neg |  | Pos |  | Pos | Pos |  |  |  |  |  |  |
| 117 | N/A | Yes | Yes | Yes | Neg | Neg | Neg | Neg |  | Neg | Neg | Neg | Neg |  |  |  |  |  |  |
| 118 | N/A | Yes | No | Yes | Pos | Neg | Pos | Neg |  | Pos | Pos | Pos | Pos |  |  |  |  |  |  |
| 119 | N/A | No | No | Yes | Neg | Neg | Neg | Neg |  | Pos | Pos | Pos | Pos |  |  |  |  |  |  |
| 120 | N/A | Yes | No | Yes | Pos |  | Pos | Neg |  | Pos |  | Pos | Pos |  |  |  |  |  |  |
| 121 | N/A | Yes | Yes | No | Pos | Neg | Pos | N/A |  | Pos | Pos | Pos | N/A |  |  |  |  |  |  |
| 122 | 1 | N/A | Yes | No | Pos |  | Pos | Pos |  | Neg |  | Neg | Neg |  | Yes | Yes | Yes |  | KP276/THA/  2018 |
| 123 | N/A | N/A | Yes | No | Pos | Neg | Pos | Neg |  | Neg | Neg | Neg | Neg |  |  |  |  |  |  |
| 124 | N/A | N/A | Yes | Yes | Pos | Pos | Neg |  |  | Pos | Pos | Neg |  |  |  |  |  |  |  |
| 125 | N/A | No | Yes | Yes | Pos | Neg | Pos |  |  | Neg | Neg | Neg |  |  |  |  |  |  |  |
| 126 | 28 | Yes | No | No | Pos |  | Pos | Pos |  | Neg |  | Neg | Neg |  |  | Yes |  |  | KP285/THA/  2018 |
| 127 | N/A | Yes | No | Yes | Pos |  | Pos | Neg |  | Neg |  | Neg | Neg |  |  |  |  |  |  |
| 128 | N/A | Yes | No | No | Neg |  |  | Neg |  | Neg |  |  | Neg |  |  |  |  |  |  |
| 129 | N/A | Yes | No | Yes | Neg | Neg | Neg | Neg |  | Neg | Neg | Neg | Neg |  |  |  |  |  |  |
| 130 | N/A | Yes | Yes | No | Pos | Neg | Pos | Neg |  | Neg | Neg | Neg | Neg |  |  |  |  |  |  |
| 131 | 1 | Yes | No | Yes | Neg | Neg | Neg | Neg |  | Neg | Neg | Neg | Neg |  |  |  |  |  |  |
| 132 | N/A | Yes | No | No | Neg | Neg | Neg | Neg |  | Neg | Neg | Neg | Neg |  |  |  |  |  |  |
| 133 | N/A | Yes | No | Yes | Neg | Neg | Neg | Neg |  | Neg | Neg | Neg | Neg |  |  |  |  |  |  |
| 134 | N/A | Yes | No | No | Neg |  | Neg |  |  | Pos |  | Pos |  |  |  |  |  |  |  |
| **Cat no.^1^** | **Age (Month)** | **Vaccination history^2^** | **Clinical sign presentations^1^** | | **RT-qPCR result^3^** | **Sample types^4^** | | | | **qPCR result^3^** | **Sample types^4^** | | | | **Sequencing^5^** | | | | |
|  |  |  | **Oral** | **Respiratory** | **FCV** | **NS** | **OS** | **RS** | **FT** | **FHV-1** | **NS** | **OS** | **RS** | **FT** | **VP1** | **VP2** | **RdRP** | **WGS** | **Strain’s name** |
| 135 | 42 | No | No | Yes | Neg | Neg | Neg |  |  | Neg | Neg | Neg |  |  |  |  |  |  |  |
| **136** | 12 | Yes | No | No | Neg |  | Neg | Neg |  | Pos |  | Pos | Neg |  |  |  |  |  |  |
| 137 | 8 | Yes | No | Yes | Neg |  | Neg |  |  | Neg |  | Neg |  |  |  |  |  |  |  |
| 138 | 48 | Yes | No | Yes | Neg |  | Neg |  |  | Neg |  | Neg |  |  |  |  |  |  |  |
| 139 | 36 | Yes | No | Yes | Neg |  | Neg |  |  | Neg |  | Neg |  |  |  |  |  |  |  |
| 140 | 60 | Yes | Yes | Yes | Pos |  | Pos |  |  | Pos |  | Pos |  |  | Yes |  | Yes |  | KP313/THA/  2019 |
| 141 | 14 | Yes | Yes | Yes | Neg |  | Neg |  |  | Pos |  | Pos |  |  |  |  |  |  |  |
| 142 | 14 | Yes | Yes | Yes | Neg |  | Neg |  |  | Pos |  | Pos |  |  |  |  |  |  |  |
| **143** | 5 | No | No | No | Neg |  | Neg |  |  | Neg |  | Neg |  |  |  |  |  |  |  |
| 144 | 14 | Yes | No | Yes | Neg | Neg | Neg |  |  | Pos | Neg | Pos |  |  |  |  |  |  |  |
| 145 | 7 | No | No | Yes | Neg | Neg | Neg | Neg |  | Pos | Neg | Pos | Neg |  |  |  |  |  |  |
| 146 | 60 | Yes | No | Yes | Neg | Neg | Neg | Neg |  | Neg | Neg | Neg | Neg |  |  |  |  |  |  |
| 147 | 72 | Yes | No | Yes | Neg | Neg | Neg | Neg |  | Neg | Neg | Neg | Neg |  |  |  |  |  |  |
| 148 | 145 | Yes | No | Yes | Pos | Neg | Pos | Neg |  | Neg | Neg | Neg | Neg |  | Yes | Yes |  |  | KP331/THA/  2020 |
| 149 | 60 | Yes | No | Yes | Neg | Neg | Neg |  |  | Neg | Neg | Neg |  |  |  |  |  |  |  |
| 150 | 120 | Yes | No | Yes | Neg | Neg | Neg | Neg |  | Neg | Neg | Neg | Neg |  |  |  |  |  |  |
| 151 | 16 | Yes | No | Yes | Pos | Pos | Neg | Neg |  | Neg | Neg | Neg | Neg |  | Yes | Yes |  |  | KP339/THA/  2020 |
| 152 | 11 | Yes | No | Yes | Neg | Neg | Neg | Neg |  | Neg | Neg | Neg | Neg |  |  |  |  |  |  |
| 153 | 24 | N/A | No | Yes | Neg | Neg | Neg | Neg |  | Pos | Pos | Pos | Pos |  |  |  |  |  |  |
| 154 | 36 | Yes | No | Yes | Pos | Neg | Pos | Neg |  | Neg | Neg | Neg | Neg |  |  | Yes |  |  | KP351/THA/  2021 |
| 155 | 8 | No | Yes | No | Pos | Neg | Pos | Neg |  | Neg | Neg | Neg | Neg |  |  | Yes |  |  | KP354/THA/  2021 |
| 156 | 6 | No | No | Yes | Neg | Neg | Neg | Neg |  | Pos | Pos | Pos | Neg |  |  |  |  |  |  |
| **Cat no.^1^** | **Age (Month)** | **Vaccination history^2^** | **Clinical sign presentations^1^** | | **RT-qPCR result^3^** | **Sample types^4^** | | | | **qPCR result^3^** | **Sample types^4^** | | | | **Sequencing^5^** | | | | |
|  |  |  | **Oral** | **Respiratory** | **FCV** | **NS** | **OS** | **RS** | **FT** | **FHV-1** | **NS** | **OS** | **RS** | **FT** | **VP1** | **VP2** | **RdRP** | **WGS** | **Strain’s name** |
| 157 | 8 | Yes | Yes | Yes | Pos |  | Pos |  |  | Neg |  | Neg |  |  | Yes | Yes |  |  | KP365/THA/  2021 |
| 158 | 36 | Yes | No | Yes | Pos | Pos | Pos |  |  | Pos | Pos | Pos |  |  |  |  |  |  |  |
| 159 | 6 | Yes | No | Yes | Neg | Neg | Neg |  |  | Neg | Neg | Neg |  |  |  |  |  |  |  |
| 160 | N/A | Yes | No | Yes | Neg | Neg | Neg |  |  | Pos | Pos | Pos |  |  |  |  |  |  |  |
| 161 | N/A | Yes | No | Yes | Neg | Neg | Neg |  |  | Pos | Pos | Pos |  |  |  |  |  |  |  |
| 162 | N/A | Yes | No | Yes | Neg | Neg | Neg |  |  | Neg | Neg | Neg |  |  |  |  |  |  |  |
| 163 | N/A | Yes | Yes | No | Neg | Neg | Neg |  |  | Neg | Neg | Neg |  |  |  |  |  |  |  |
| 164 | 48 | Yes | No | Yes | Pos |  |  |  | Pos | Pos |  |  |  | Pos |  |  |  |  |  |
| 165 | 3 | No | No | Yes | Pos |  |  |  | Pos | Pos |  |  |  | Pos |  |  |  | Yes | KP361/THA/  2021 |
| 166 | 24 | N/A | No | No | Neg |  |  |  | Neg | Pos |  |  |  | Pos |  |  |  |  |  |
| 167 | 60 | N/A | No | No | Neg |  |  |  | Neg | Pos |  |  |  | Pos |  |  |  |  |  |
| 168 | 24 | N/A | No | No | Neg |  |  |  | Neg | Neg |  |  |  | Neg |  |  |  |  |  |
| 169 | 1 | No | Yes | Yes | Pos |  |  |  | Pos | Neg |  |  |  | Neg |  |  |  |  |  |
| 170 | 6 | N/A | No | No | Neg |  |  |  | Neg | Neg |  |  |  | Neg |  |  |  |  |  |
| 171 | 6 | N/A | No | No | Neg |  |  |  | Neg | Pos |  |  |  | Pos |  |  |  |  |  |
| 172 | 6 | N/A | No | No | Neg |  |  |  | Neg | Pos |  |  |  | Pos |  |  |  |  |  |
| 173 | 4 | No | No | No | Pos |  |  |  | Pos | Neg |  |  |  | Neg |  |  |  |  |  |
| 174 | N/A | N/A | No | No | Neg |  |  |  | Neg | Neg |  |  |  | Neg |  |  |  |  |  |
| 175 | N/A | N/A | No | No | Neg |  |  |  | Neg | Neg |  |  |  | Neg |  |  |  |  |  |
| 176 | 4 | No | Yes | Yes | Pos |  |  |  | Pos | Neg |  |  |  | Neg |  | Yes |  |  | KP374/THA/  2021 |
| 177 | 12 | Yes | No | No | Neg |  |  |  | Neg | Pos |  |  |  | Pos |  |  |  |  |  |
| 178 | N/A | N/A | No | No | Neg |  |  |  | Neg | Pos |  |  |  | Pos |  |  |  |  |  |
| 179 | N/A | N/A | No | No | Neg |  |  |  | Neg | Pos |  |  |  | Pos |  |  |  |  |  |
| **Cat no.^1^** | **Age (Month)** | **Vaccination history^2^** | **Clinical sign presentations^1^** | | **RT-qPCR result^3^** | **Sample types^4^** | | | | **qPCR result^3^** | **Sample types^4^** | | | | **Sequencing^5^** | | | | |
|  |  |  | **Oral** | **Respiratory** | **FCV** | **NS** | **OS** | **RS** | **FT** | **FHV-1** | **NS** | **OS** | **RS** | **FT** | **VP1** | **VP2** | **RdRP** | **WGS** | **Strain’s name** |
| **180** | N/A | N/A | No | No | Neg |  |  |  | Neg | Pos |  |  |  | Pos |  |  |  |  |  |
| **181** | N/A | N/A | No | No | Neg |  |  |  | Neg | Pos |  |  |  | Pos |  |  |  |  |  |
| **182** | N/A | N/A | No | No | Neg |  |  |  | Neg | Pos |  |  |  | Pos |  |  |  |  |  |
| **183** | N/A | N/A | No | No | Neg |  |  |  | Neg | Pos |  |  |  | Pos |  |  |  |  |  |
| **184** | N/A | N/A | No | Yes | Neg |  |  |  | Neg | Pos |  |  |  | Pos |  |  |  |  |  |

^1^ Healthy cats presented in bold (n=53), while FURTD and/or oral disease affected cats (n=131) showed signs of oculonasal discharge, sneezing, coughing and gingivostomatitis. The missing data means no data available.

^2^ Vaccination for feline rhinotracheitis virus (caused by FCV and FHV-1) within one year before sampling.

^3^ Reverse transcription real-time polymerase chain reaction (RT-qPCR) result for FCV and PCR result for FHV-1: Pos (positive); Neg (negative)

^4^ Sample types for molecular detection: NS (nasal swab); OS (oropharyngeal swab); RS (rectal swab); FT (homogenized fresh tissue; n=21)

^5^ Cats achieving full-length VP1 major capsid protein gene (n=14), full-length VP2 minor capsid protein gene (n=12), partial RdRp gene (n=12), and whole genome sequence (WGS; n=1).

N/A = Not applicable
