# Supplementary material for: Molecular epidemiology and strain diversity of circulating feline Calicivirus in Thai cats
Source: Front Vet Sci. 2024 Jun 3;11:1377327. doi: 10.3389/fvets.2024.1377327 (PMC11180889; doi:10.3389/fvets.2024.1377327)
Supplement: Supplementary file 3 [file Image_1.pdf]

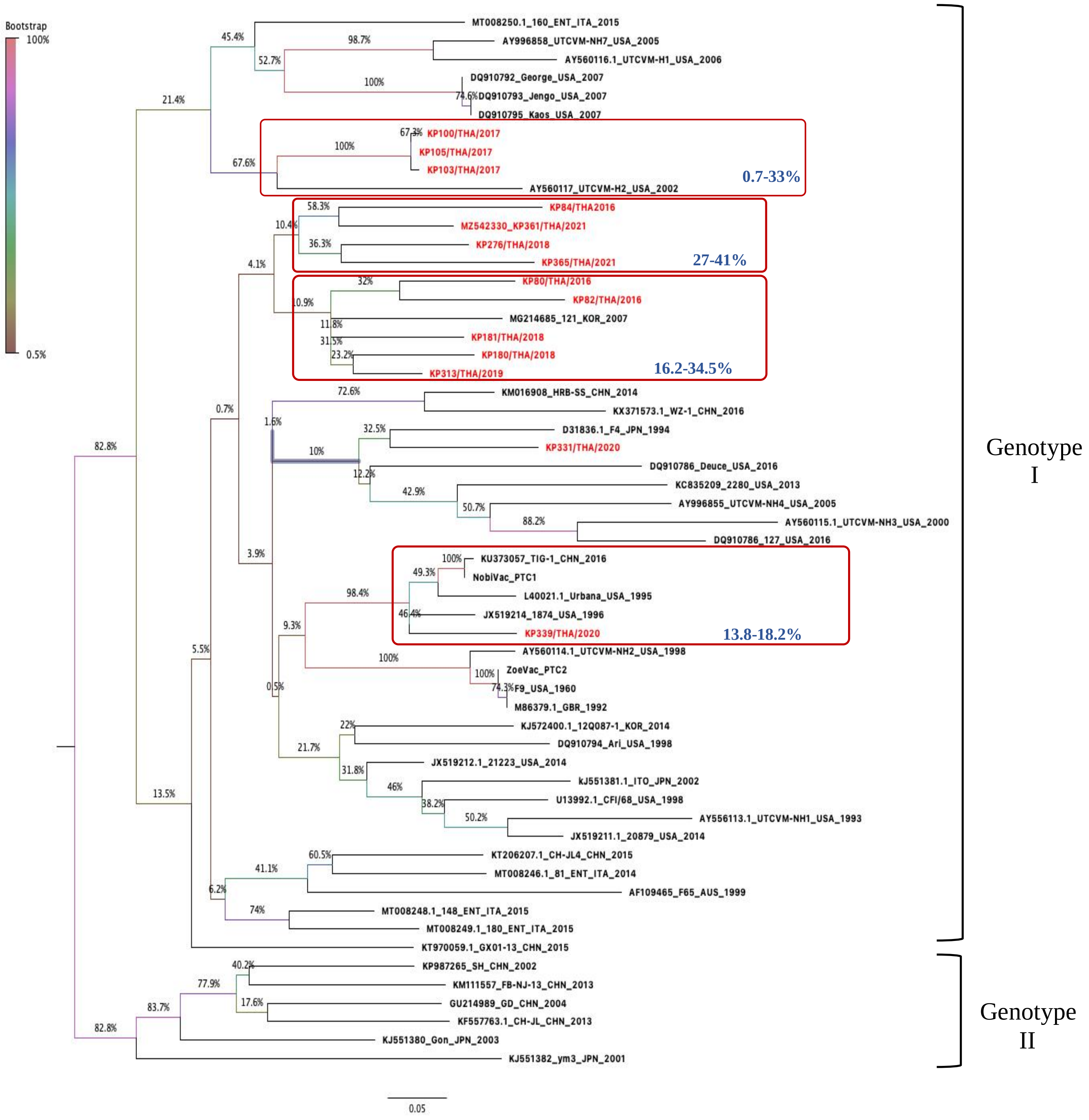

Supplementary Figure S1. Phylogenetic trees of P2 subdomain. The trees were constructed based on deduced amino acids using the maximum likelihood method with Whelen and Goldman model. The statistical support supplied with bootstrapping of 1,000 replicates and significantly considered when a bootstrap value is greater than 70. FCV-TH strains were presented in red letters. The pairwise p-distance of each group was shown in each box by a limited threshold as < 20 percent divergence for a single strain.

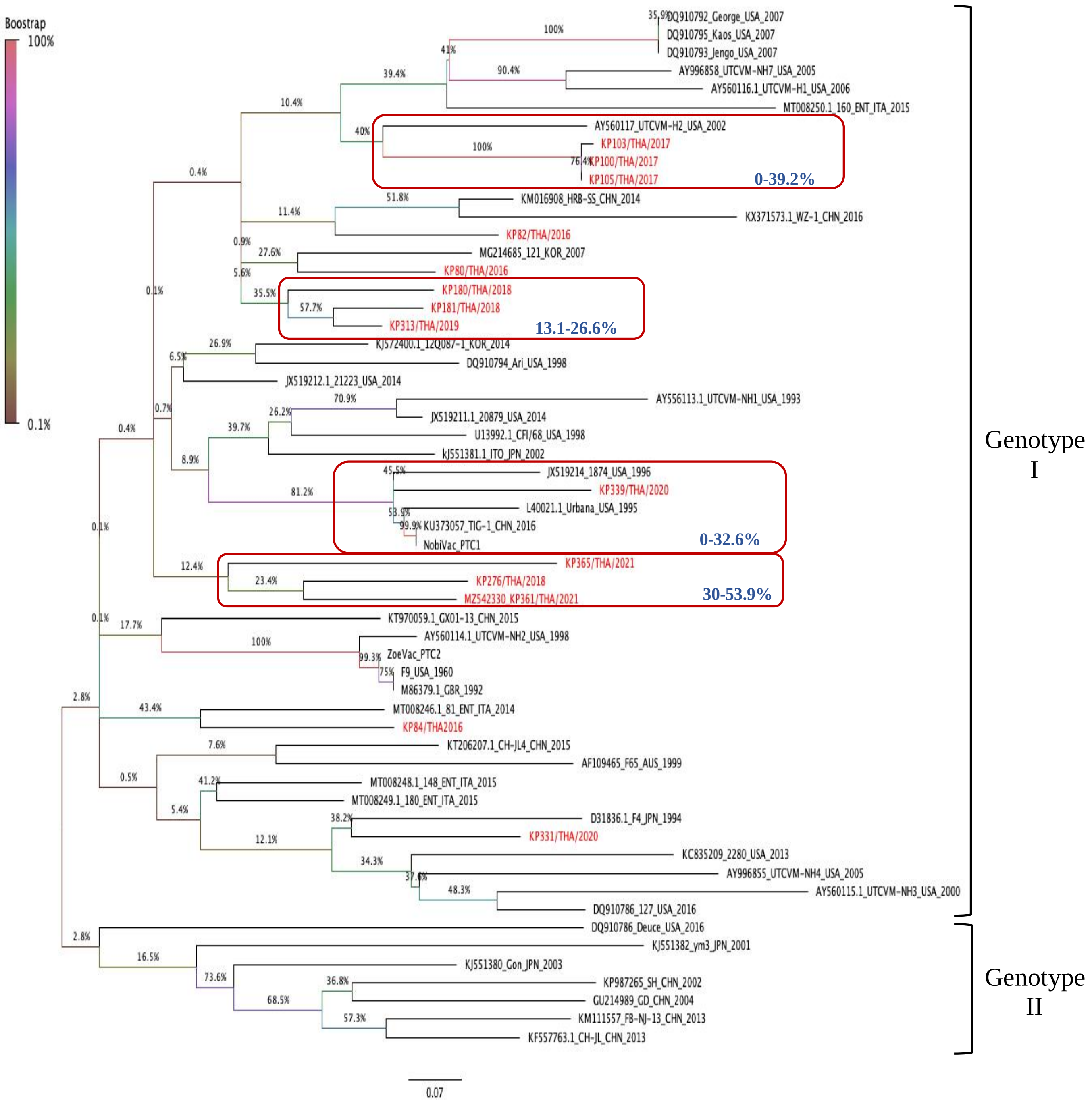

Supplementary Figure S2. Phylogenetic trees of hypervariable E region (HVR-E). The trees were constructed based on deduced amino acids using the maximum likelihood method with Le Gascuel 2008 model. The statistical support supplied with bootstrapping of 1,000 replicates and significantly considered when a bootstrap value is greater than 70. FCV-TH strains were presented in red letters. The pairwise p-distance of each group was shown in each box by a limited threshold as < 20 percent divergence for a single strain.
